# Association between tomoelastography and histological grade and clinical characteristics of pancreatic neuroendocrine neoplasms

**DOI:** 10.1093/gastro/goag007

**Published:** 2026-03-14

**Authors:** Yu-Qing Zhong, Xi-Tai Huang, Yan-Ji Luo, Chen-Song Huang, Qiong-Cong Xu, Xiao-Yu Yin

**Affiliations:** Department of Pancreato-Biliary Surgery, The First Affiliated Hospital, Sun Yat-sen University, Guangzhou, Guangdong, P. R. China; Department of Pancreato-Biliary Surgery, The First Affiliated Hospital, Sun Yat-sen University, Guangzhou, Guangdong, P. R. China; Department of Radiology, The First Affiliated Hospital, Sun Yat-sen University, Guangzhou, Guangdong, P. R. China; Department of Pancreato-Biliary Surgery, The First Affiliated Hospital, Sun Yat-sen University, Guangzhou, Guangdong, P. R. China; Department of Pancreato-Biliary Surgery, The First Affiliated Hospital, Sun Yat-sen University, Guangzhou, Guangdong, P. R. China; Department of Pancreato-Biliary Surgery, The First Affiliated Hospital, Sun Yat-sen University, Guangzhou, Guangdong, P. R. China

**Keywords:** pancreatic neuroendocrine neoplasm, tomoelastography, histological grade, tissue stiffness

## Abstract

**Background:**

The prognosis of a pancreatic neuroendocrine neoplasm (pNEN) is closely correlated with histological grade. While the role of tomoelastography in predicting tumor grades has been explored in various cancers, evidence regarding its association with the histological grade and clinical features of pNENs remains limited. This study aimed to investigate the association between tomoelastographic parameters and the histological grade and key clinical characteristics of pNENs.

**Methods:**

A retrospective study was conducted on 62 patients with pathologically confirmed pNENs, all of whom underwent tomoelastography prior to surgery without receiving neoadjuvant treatment. Patients were categorized into three groups: G1 (*n *= 28), G2 (*n *= 30), and G3/neuroendocrine carcinoma (NEC) (*n *= 4). The relationship between the tomoelastography parameters and clinicopathological characteristics was analysed by using the Kruskal–Wallis test, Spearman correlation, and ordinal logistic regression. Receiver-operating characteristic curves were used for evaluating the diagnostic performance of tomoelastography.

**Results:**

The shear wave speed (*c*), representing stiffness in tomoelastography, increased with tumor grade (1.63 m/s for G1 vs 2.23 m/s for G2 vs 2.53 m/s for G3&NEC, *P *< 0.001). Parameter *c* was positively correlated with the tumor size (*r *= 0.59, *P *< 0.001) and Ki67 index (*r *= 0.44, *P *< 0.001), and was notably higher in lesions with distant or regional lymph node metastases than in those without metastases. Identified as a hazardous factor for tumor grade (odds ratio = 3.92, 95% confidential interval [CI]: 1.88–8.16), *c* showed good performance in discriminating between G1 and G2 (area under the curve = 0.81, 95% CI: 0.70–0.93, *P *< 0.001).

**Conclusion:**

Tomoelastography offers a promising quantitative tool for assessing the histological grade of pNENs and identifying more aggressive tumor behavior via increased tissue stiffness.

## Introduction

Pancreatic neuroendocrine neoplasm (pNEN) is a rare type of tumor characterized by neuroendocrine differentiation and the expression of neuroendocrine markers [1]. In the past 40 years, with the advances in diagnostic technology, the incidence of pNENs has increased significantly [2].

The biological behavior of pNENs is highly heterogeneous, ranging from indolent growth to aggressive invasion and early metastasis [3, 4]. Their biological characteristics can change as the disease progresses. pNENs can occur sporadically or be genetically linked and can be categorized as functional or nonfunctional based on hormone production. It is recommended to use the latest version of the World Health Organization (WHO) classification, released in 2022, to grade pNENs based on their tissue differentiation and cell proliferation activity [5]. Cell proliferation activity is measured by using the mitotic count and Ki-67 index. The prognosis and treatment strategies for pNENs vary significantly based on the histological grade and stage [6, 7]. Patients with higher WHO grades or metastases generally have worse outcomes [8]. Therefore, preoperative assessment of the tumor grade is essential for treatment planning and prognosis evaluation.

In recent years, growing attention has been devoted to exploring the biological properties of both the tumor microenvironment (TME) and the tumor itself. Mechanical factors—such as solid stress, interstitial fluid pressure, tissue stiffness, and the composition of the peritumoral matrix—are increasingly recognized as important drivers of tumor growth, progression, metastasis, and therapeutic response [9, 10]. Importantly, they may serve as valuable diagnostic and prognostic biomarkers by capturing biological features that cannot be assessed through conventional imaging. Tomoelastography, which is based on multifrequency magnetic resonance elastography (MRE), is a novel imaging technique to measure tissue stiffness [11] and offers a unique opportunity to noninvasively assess these biomechanical alterations. Tomoelastography provides two mechanical parameters: shear wave speed (*c*, m/s) and phase angle (*φ*, rad), which are surrogate markers for stiffness and fluidity, respectively [12]. With the development of radiology, several studies have explored the potential of noninvasive methods, such as computed tomography, magnetic resonance imaging, positron emission tomography, ultrasound, and artificial intelligence, to predict the histological grade of pNENs [13]. Tomoelastography provides a novel method to assess the histological grade of pNENs with demonstrated repeatability and reproducibility in pancreas imaging [14, 15]. Several studies have highlighted the value of MRE in clinical practice for assessing hepatic fibrosis [16], detecting hepatocellular carcinomas [17], evaluating pancreatic cancer [18], determining tumor aggressiveness in rectal cancer [19], and predicting postoperative pancreatic fistula [20]. Currently, there is limited research evaluating the role of tomoelastography in predicting the tumor grade and clinical stage in pNENs.

This study aimed to evaluate the value of tomoelastography in predicting the WHO grade of pNENs, offering a noninvasive method for comprehensive assessment.

## Methods

### Patients

Patients with pathologically confirmed pNENs at the First Affiliated Hospital of Sun Yat-sen University (Guangzhou, China) from 1 June 2021 to 30 April 2025 were retrospectively included. The inclusion criteria were as follows: (i) patients with pNENs proven by using postoperative pathology, (ii) preoperative tomoelastography performed within 30 days before surgery, and (iii) no neoadjuvant treatment before tomoelastography examination, including somatostatin analogs, chemotherapy, or targeted therapy. The exclusion criteria were as follows: (i) patient with severe pancreatic atrophy resulting in lack of region of interest (ROI) in elastogram or (2) no available images or poor images.

### Clinical and histopathological characteristics

Clinical data, including age, gender, tumor location, surgery, and postoperative treatment, were collected. The tumor stage of all patients was determined according to the American Joint Committee on Cancer Staging Manual, 8th Edition; the Tumor-Node-Metastasis (TNM) staging system assesses the extent of cancer based on the primary tumor (T), the involvement of regional lymph nodes (N), and the presence of distant metastasis (M) [21]. Histopathological analysis was conducted on surgical specimens to assess the tumor size, WHO grade, lymph node status, and resection margin. The tumor grade was determined according to the 2022 WHO classification [5].

### Tomoelastography examination

The protocol for imaging acquisition and post-processing methods has been illustrated in a previous study [15]. Multifrequency wave-field data were acquired over a period of 7 minutes and 22 seconds by using a single-shot spin-echo echo-planar imaging sequence equipped with flow-compensated motion-encoding gradients (MEGs). The MEGs were applied at an amplitude of 45 mT/m. Vibration frequencies of 30.00, 40.00, 50.00, and 60.00 Hz were applied, corresponding to MEG frequencies of 37.20, 37.20, 37.48, and 44.88 Hz, respectively. Tomoelastography data were reconstructed by using the publicly available multifrequency MRE pipeline (https://bioqic-apps.com). A multifrequency wave-number-based algorithm generated high-resolution, full-field maps. The shear wave speed (*c*) was directly proportional to the square root of the complex shear modulus, which can be considered a surrogate marker for stiffness. The phase angle of the complex shear modulus (*φ*) images was generated by using the Laplacian operators-based processing method, reflecting the fluidity properties of the tissue [12]. Two radiologists with experience in abdominal magnetic resonance imaging analysed the images by using ImageJ software (version 1.51, National Institute of Health, USA). The radiologists adjusted the brightness and contrast of the images and selected the clearest and largest pancreatic slices on the *c* map and *φ* map. ROIs on the *c* map were manually drawn to measure the stiffness within the solid-tumor portion, excluding calcifications, blood vessels, necrosis, cystic areas, and pancreatic duct dilation, and were then copied to the *φ* map to measure the fluidity.

### Statistical methods

The continuous data were tested for normality by using the Shapiro–Wilk test. The normally distributed data are presented as means with standard deviations and the nonparametric data are presented as medians with interquartile ranges (IQRs). The chi-square test, analysis of variance (ANOVA), or Kruskal–Wallis test was performed to compare clinical data (gender, age, etc.). Differences between different grade groups were calculated by using the Kruskal–Wallis test for nonparametric data or the least significant difference tests for normally distributed data. Spearman’s correlation was used to assess the relationships between the tomoelastography parameters, tumor size, and WHO grade.

The ordinal logistic regression analysis was performed to identify independent risk factors for the WHO grade. Receiver-operating characteristic (ROC) curves were created to evaluate the diagnostic performance of the tomoelastography in discriminating between the different WHO grades. Statistical analyses were performed by using SPSS 25.0 (IBM, NY, USA). Plots were generated by using GraphPad Prism (version 9.0, San Diego, CA, USA). A two-sided *P* value of <0.05 was considered statistically significant.

## Results

### Population demographics and histopathological features

A total of 62 pNEN cases were included in the study (mean age, 52.4 ± 11.7 years), consisting of 26 males and 36 females. The tumor locations were as follows: 28 lesions in the head or neck of the pancreas and 34 in the body or tail. Histologically, 28 (45.2%) were G1, 30 (48.4%) were G2, 2 (3.2%) were G3, and 2 (3.2%) were neuroendocrine carcinoma (NEC). The population demographics are presented in Table 1.

**Table 1:** Population demographics of 62 patients with pNENs included in this study

Parameter	No. of patients (%)
Age, years, mean ± SD	52.4 ± 11.7
Gender (males/females)	26/36
Location	
Head–neck	28 (45.2)
Body–tail	34 (54.8)
Neoplasm type	
Nonfunctional	54 (87.1)
Insulinoma	6 (9.7)
Gastrinoma	2 (3.2)
Grade	
G1	28 (45.2)
G2	30 (48.4)
G3	2 (3.2)
NEC	2 (3.2)
Surgery	
Enucleation	12 (19.4)
Pancreatoduodenectomy	17 (27.4)
Total pancreatectomy	1 (1.6)
Distal pancreatectomy	31 (50.0)
Central pancreatectomy	1 (1.6)

### Tomoelastography parameters of different histological grades and TNM stages

**Figure 1.**
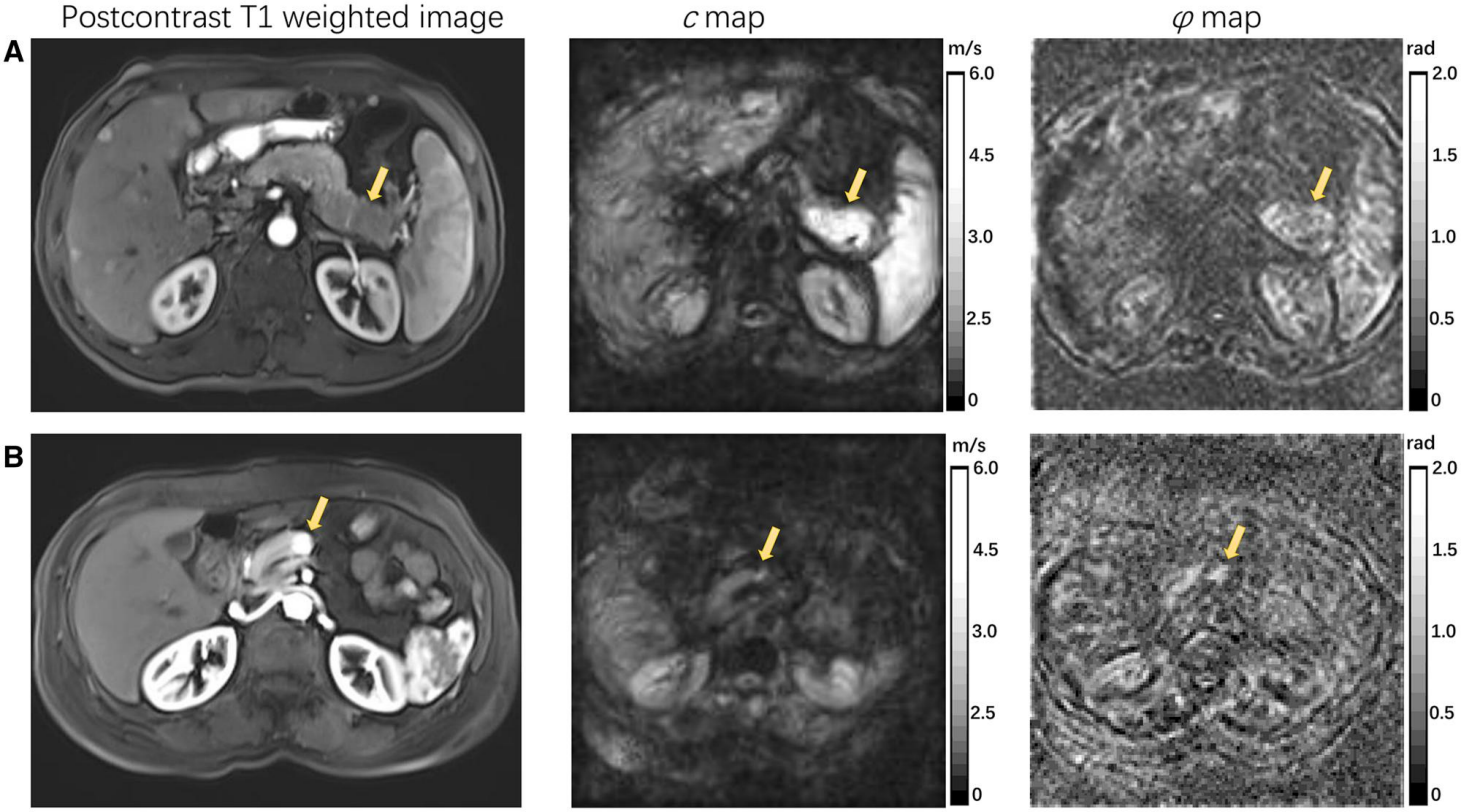
Representative axial T1-weighted images and axial tomoelastography *c* (stiffness) and *φ* (fluidity) maps for two participants. (A) Images in a participant with G3 pNEN (arrows; *c*, 5.92 m/s; *φ*, 1.23 rad). (B) Images in a participant with G1 pNEN (arrows; *c*, 1.85 m/s; *φ*, 1.14 rad).

**Figure 2.**
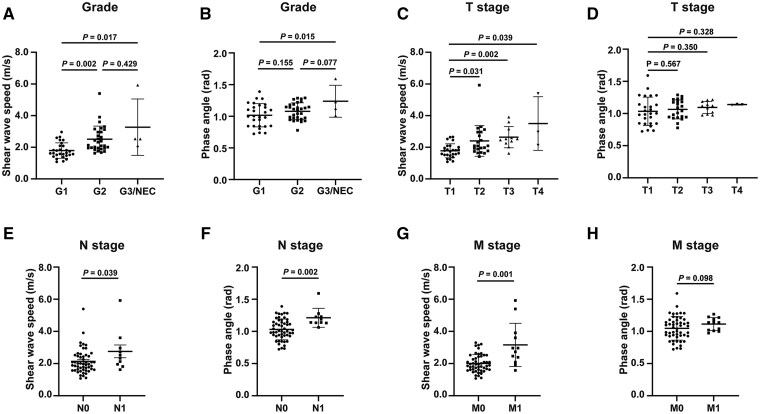
Shear wave speed and phase angle in different subgroups. (A) Shear wave speed in different histological grades. (B) Phase angle in different histological grades. (C) Shear wave speed in different T stages. (D) Phase angle in different T stages. (E) Shear wave speed in different N stages. (F) Phase angle in different N stages. (G) Shear wave speed in different M stages. (H) Phase angle in different M stages.

Figure 1 shows representative elastograms of the *c* map and *φ* map along with corresponding magnetic resonance images of the pNEN participants with different WHO grades. Table 2 presents the characteristics of patients with different WHO grades. In our study, pNENs with a higher grade (G2, G3&NEC) exhibited greater stiffness, as reflected by the significantly higher *c* values than for those with G1 lesions (G1 vs G2, *P *= 0.002; G1 vs G3, *P *= 0.017), but the *c* values did not show a significant difference between the G2 and G3&NEC groups (*P *= 0.429). Figure 2A shows the scatters of each case in different grades, with median and IQR bars. With respect to fluidity (*φ*), G3 lesions had significantly higher *φ* values than G1 lesions (*P *= 0.015) and higher *φ* values than G2 lesions at borderline significance (*P *= 0.077). Figure 2B shows the scatters of each case in different grades with mean and standard error bars.

**Table 2:** Characteristics of different grade of pNENs

Parameter	G1 (*n *= 28)	G2 (*n *= 30)	G3&NEC (*n *= 4)	*P*
Age, years, mean ± SD	52.9 ± 13.0	51.6 ± 11.1	55.3 ± 8.0	0.818
No. of males/no. of females	14/14	9/21	3/1	0.120
Tumor size, mm (range)	16.0 (12.0–24.0)	28.5 (23.50–39.3)	36.0 (22.50–87.8)	0.001
Location, *n* (%)				0.443
Head–neck	11 (39.3)	14 (46.7)	3 (75.0)	
Body–tail	17 (60.7)	16 (53.3)	1 (25.0)	
Neoplasm type, *n* (%)				0.338
Nonfunctional	22 (78.6)	28 (93.4)	4 (100)	
Insulinoma	5 (17.8)	1 (3.3)	0 (0)	
Gastrinoma	1 (3.6)	1 (3.3)	0 (0)	
CA199 (range)	5.78 (3.81–10.82)	5.78 (3.57–9.81)	19.04 (5.17–52.79)	0.322
T stage, *n* (%)				<0.001
T1	20 (71.4)	5 (16.7)	1 (25.0)	
T2	3 (10.7)	17 (56.7)	2 (50.0)	
T3	4 (14.3)	6 (20.0)	1 (25.0)	
T4	1 (3.6)	2 (6.6)	0 (0)	
N stage, *n* (%)				0.156
N0	25 (89.3)	25 (83.3)	2 (50.0)	
N1	3 (10.7)	5 (16.7)	2 (50.0)	
M stage, *n* (%)				0.104
M0	25 (89.3)	23 (76.7)	2 (50.0)	
M1	3 (10.7)	7 (23.3)	2 (50.0)	
Vascular involvement, *n* (%)	5 (17.9)	3 (10.0)	1 (25.0)	0.425
Ki-67 index, *n* (%)				<0.001
<3%	27 (96.4)	3 (10.0)	0 (0)	
3%–20%	1 (3.6)	27 (90.0)	0 (0)	
>20%	0 (0)	0 (0)	4 (100)	
*c*, m/s (range)	1.63 (1.48–2.09)	2.23 (1.95–3.00)	2.53 (2.20–5.08)	<0.001
*φ*, rad, mean ± SD	1.02 ± 0.18	1.08 ± 0.13	1.24 ± 0.25	0.038
Surgery, *n* (%)				0.009
Radical	28 (100)	29 (96.7)	2 (50.0)	
Debulking	0 (0)	1 (0.3)	2 (50.0)	

Analysis by using the T stage revealed a progressive increase in the stiffness from T1 to more advanced T stages. The T2, T3, and T4 groups had significantly higher *c* values than did the T1 group (T1: 1.76 [1.52–2.01] m/s vs T2: 2.09 [1.77–2.96] m/s, *P *= 0.031; vs T3: 2.53 [2.32–2.88] m/s, *P *= 0.002; vs T4: 2.96 [2.55–4.18] m/s, *P *= 0.039). In contrast, the *φ* values did not differ between the different T stages. Both the *c* and *φ* values were positively associated with regional lymph node metastasis. Lesions with regional lymph node metastasis presented higher *c* (2.42 [1.96–2.96] m/s vs 1.97 [1.62–2.50] m/s, *P *= 0.039) and *φ* (1.21 ± 0.15 rad vs 1.03 ± 0.16 rad, *P *= 0.002) values. Additionally, the *c* value was also related to the M stage: the lesions with distant metastasis had higher *c* values than did those without distant metastasis (2.85 [2.24–3.60] m/s vs 1.92 [1.63–3.75] m/s, *P *= 0.001). Figure 2C–H shows the *c* and *φ* values for different TNM stages.

### Correlation between tomoelastography parameters and histopathological and clinical characteristics

**Figure 3.**
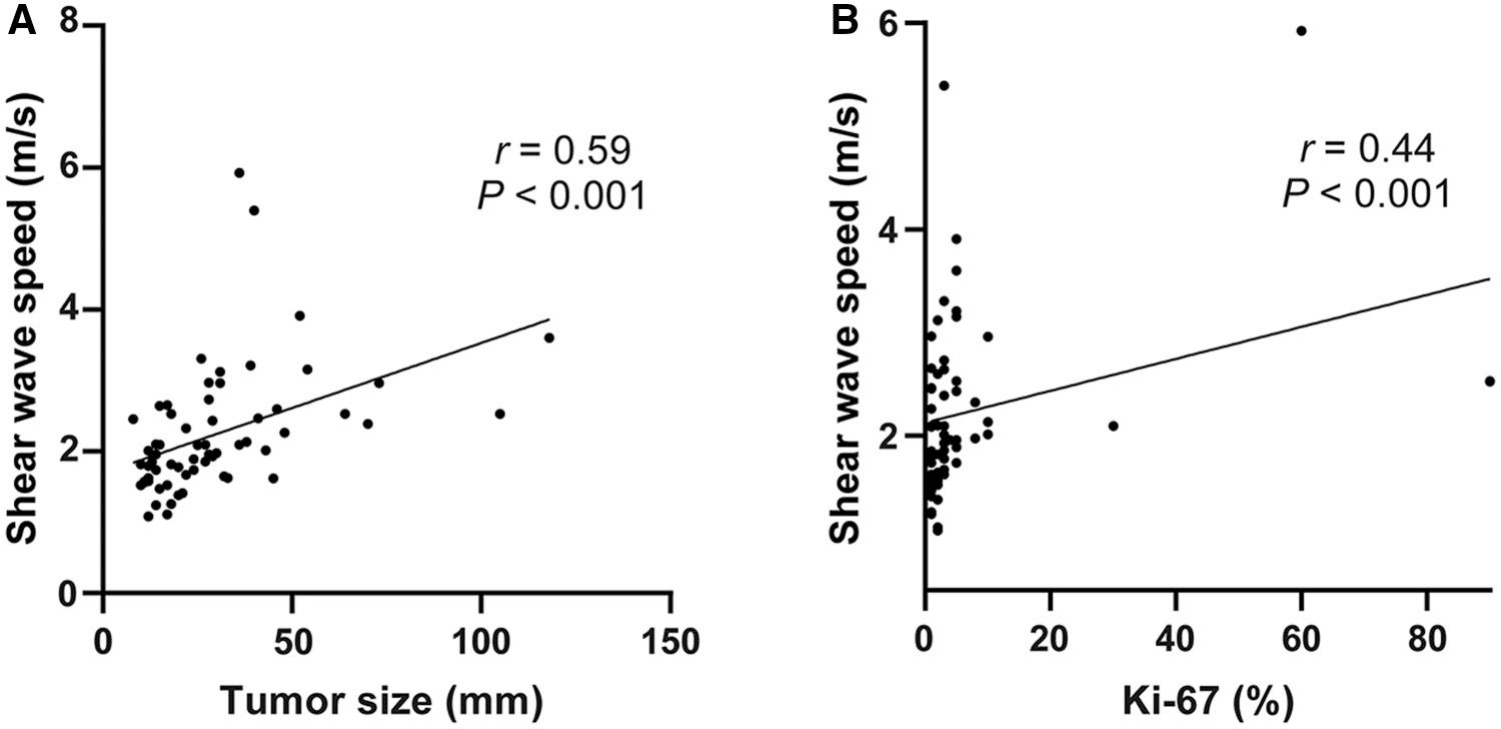
Correlation scatter plot of tomoelastography parameters and tumor size. (A) Correlation scatter plot of shear wave speed and tumor size. (B) Correlation scatter plot of shear wave speed and Ki67 index.

According to Spearman correlation analysis, the *c* value showed a positive correlation with the WHO grade (*r *= 0.56, *P *< 0.001), Ki67 index (*r *= 0.44, *P *< 0.001), and tumor size (*r *= 0.59, *P *< 0.001). After adjustment for tumor size by using partial correlation, the *c* value remained positively correlated with the WHO grade (*r *= 0.41, *P *= 0.001). Figure 3 shows scatter plots of the *c* values with the tumor size and Ki67 index.

### Ordinal regression and evaluation

**Figure 4.**
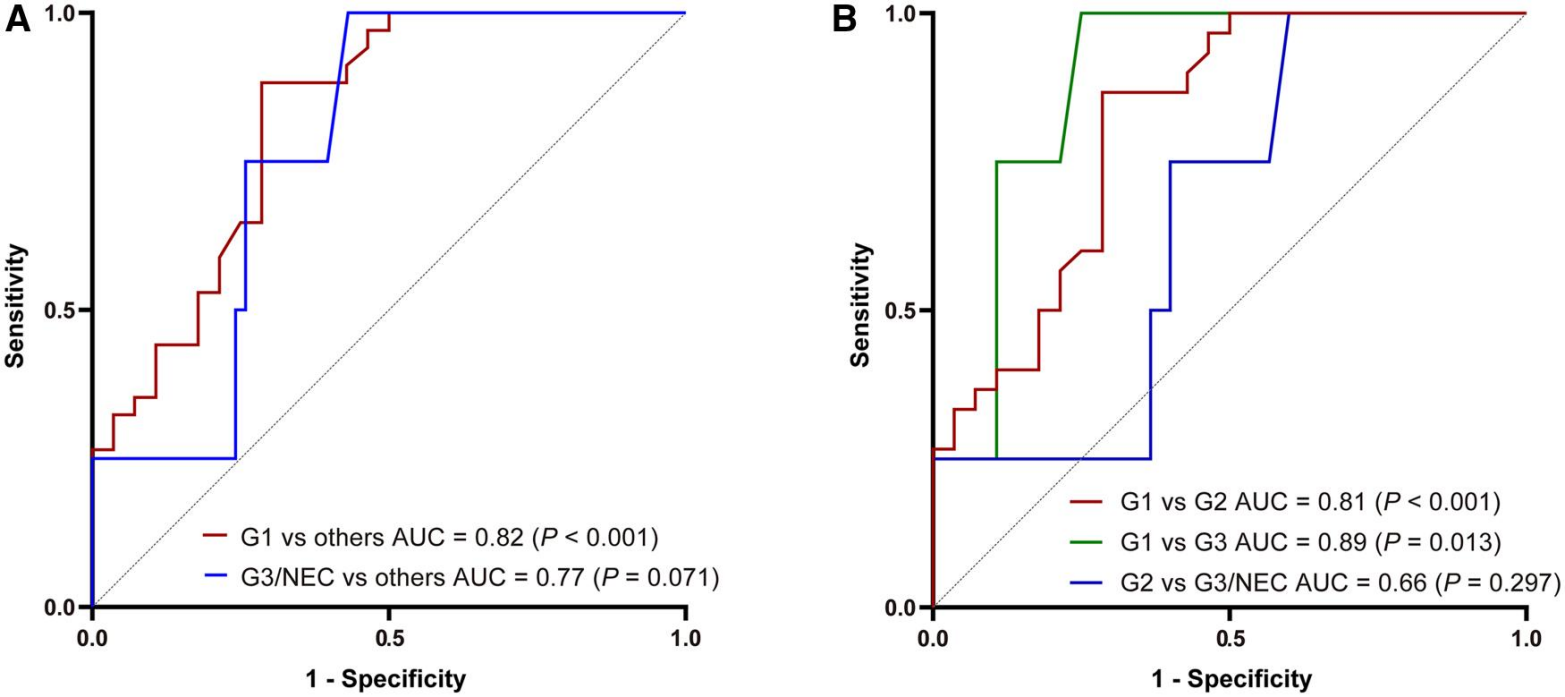
ROC curves of *c* values in discriminating between pNENs with different WHO grades. (A) ROC curves of *c* values in distinguishing G1 from other higher grades and G3&NEC from other lower grades. (B) ROC curves of *c* values in discriminating between G1 and G2, G1 and G3&NEC), and G2 and G3&NEC.

Ordinal logistic regression identified the *c* value as a significant predictor of the WHO grade (odds ratio [OR] = 3.92 [95% CI: 1.88–8.16], *P *< 0.001), indicating that an increase in the *c* value by one unit raised the likelihood of a higher grade by 3.916 times, but the *φ* value was not a significant predictor (OR = 4.71 [95% CI: 0.14–158.44], *P *= 0.388). As shown in Figure 4A, the ROC analysis demonstrated that the *c* value had good diagnostic performance in discriminating G1 from higher grades (area under the curve [AUC] = 0.82 [95% CI: 0.72–9.93], *P *< 0.001), but did not perform well with significance in distinguishing between G3&NEC and lower grades (AUC = 0.77 [95% CI: 0.60–0.94], *P *= 0.071). Considering the limited number of cases in the G3&NEC group, we further analysed the power of the *c* value in discriminating between G1 and G2, and found that it showed good performance (AUC = 0.81 [95% CI: 0.70–0.93], *P *< 0.001) (Fig. 4B).

## Discussion

Our study revealed that the shear wave speed (*c*), representative of stiffness derived by using tomoelastography, was associated with the WHO grade and clinical stage of pNENs. pNEN lesions with aggressive patterns, such as greater tumor size, regional lymph node metastasis, or distant metastasis, exhibited higher stiffness in tomoelastography.

The WHO grade of pNENs is a critical determinant for both prognostic assessment and treatment planning. Patients with G2/G3 pNENs or NEC generally have worse prognosis and require more aggressive surgical treatment than those with G1 tumors [22]. Therefore, clarifying the grade of tumor before surgery has important guiding value for clinical decision-making and prognosis evaluation. Endoscopic ultrasound-guided fine-needle aspiration biopsy is a widely used method to obtain accurate grading preoperatively [23]. However, its limitations include technical failures in small lesions, sampling bias due to tumor heterogeneity [24], and the risk of needle tract seeding [25]. These challenges highlight the clinical need for reliable, noninvasive approaches to predict tumor grade.

The changes in the mechanical properties of the TME are caused by the remodeling of the extracellular matrix (ECM) by tumor cells or activated stromal cells. An increase in ECM deposition and cross-linking together leads to an increase in the ECM hardness, thereby affecting the phenotype of tumor cells [26]. Matrix stiffening facilitates the proliferation, invasion, and migration of tumor cells, enhances the secretion of pro-tumorigenic factors, affects antitumor immune signaling, and impairs chemotherapeutic response [27–30]. In terms of pNENs, intratumoral fibrosis has been identified as an adverse prognostic factor for recurrence-free survival and overall survival [31]. Cancer-associated fibroblasts (CAFs), as a major cluster in the TME, have recently been described in pNENs and include myofibroblastic CAFs (myCAFs), inflammatory CAFs (iCAFs), and antigen-presenting CAFs (apCAFs) [32]. It has been reported that myCAFs are spatially closer to tumor cells and promote epithelial mesenchymal transition and tumor growth, thereby contributing to distant metastasis [33]. Furthermore, Lai *et al.* [34] found that CAFs drive the growth and metastasis of aggressive pNENs. In stroma-rich pNENs, excessive secretion of apolipoprotein E by tumor cells remodels the tumor–stromal ratio and recruits CAFs, further facilitating tumor progression [35]. These findings suggest that alterations in the primary TME, particularly CAF-mediated fibrosis and stromal remodeling, may underlie the observed association between primary tumor stiffness and metastatic potential, providing a mechanistic basis for the indirect predictive value of tomoelastography. Gültekin *et al.* [36] first illustrated that tomoelastography-derived stiffness (*c*) and fluidity (*φ*) were abnormally increased in pNEN lesions and the *c* value was associated with greater tumor size. Consistently, we found that the *c* value was associated with the WHO grade, tumor size, regional lymph node metastasis, and distant metastasis. A higher *c* value implied a more aggressive biological pattern of pNENs.

For nonfunctional pNENs (NF-pNENs), the tumor size remains the criterion for deciding between tumor resection and surveillance. NF-pNENs with a diameter of ≥20 mm are associated with a high risk of liver metastasis and are considered for treatment according to the European Neuroendocrine Tumor Society guidelines [37]. So far, the treatment of small NF-pNENs (tumor size < 20 mm) is still controversial. Previous studies have emphasized the risk of making clinical decisions based solely on the tumor size and the importance of considering both the tumor size and the histological grade [38–41]. Aggressiveness, including lymph node metastasis and distant metastasis, has been reported in small pNENs [41–43]. Thus, besides the tumor size, personalized treatment strategies that consider the tumor grade are preferable. In our study, the *c* value still showed positive correlation with the WHO grade after controlling for the tumor size, suggesting its value in predicting the biological behavior of pNENs. The sample size of NF-pNENs with a diameter of <20 mm was limited for subgroup analysis in our study, so more cases are needed to explore the clinical significance of tomoelastography in classifying the WHO grades in small NF-pNENs.

The pseudocapsule is a pathological fibrous layer that forms around tumors via reactive extracellular matrix deposition from surrounding stromal cells, such as fibroblasts, in response to lesion growth [44]. A recent study demonstrated that an incomplete pseudocapsule was associated with more aggressive tumor behavior and poorer recurrence-free and overall survival in pNENs [45]. Although limited literature exists on how the presence or integrity of a pseudocapsule may influence tomoelastography measurements, its fibrous composition and mechanical properties could potentially affect shear wave propagation or ROI delineation. Considering both its mechanical characteristics and its clinical significance, integrating pseudocapsule assessment with tomoelastography—possibly combined with MRI contrast parameters and machine-learning techniques—may enhance preoperative evaluation and improve predictive accuracy in future studies.

Our study had limitations. First, it was a retrospective single-center study with a small sample size and unbalanced group size. As it was a high-volume medical center, most patients with pNENs, especially NEC or pNENs with metastasis, had accepted neoadjuvant therapy in other hospitals before undergoing tomoelastography examination and were excluded in this study. Second, some detailed pathological characteristics, such as vascular or perineural invasion, were not included due to >20% of the data missing in pathological reports. Additional pathological features and immunohistochemical markers should be incorporated in future studies. Third, tomoelastography was an emerging tool in our center, so the follow-up time was insufficient to collect enough events for survival analysis. With the accumulation of more cases and extended follow-up, the prognostic significance of tomoelastography in pNENs may be further elucidated.

## Conclusion

Tomoelastography provides a noninvasive means to predict the WHO grade and clinical stage in pNENs, thereby offering potential value for individualized clinical decision-making.

## Authors’ contributions

X.Y.Y. conceived and designed the project; Y.Q.Z. and X.T.H. collected and analysed the data and wrote the original manuscript; Y.J.L. provided the radiologic data; C.S.H. and Q.C.X. managed the clinical data; X.Y.Y. discussed and provided comments, and revised the manuscript. All authors read and approved the final manuscript.

## Data Availability

The data presented in this study are available from the corresponding author on reasonable request.
